# Notes and News

**Published:** 1891-05-09

**Authors:** 


### NOTES AND NEWS.

COLLECTED AND COMMUNICATED.

AT the meeting of the Royal Statistical Society on the 21st inst., Dr. Steele, of Guy's Hospital read a paper on the "Charitable Aspects of Medical Relief." He said that the large and increasing number of out-patients at the various hospitals, notwithstanding the establishment of free dispensaries, rendered it necessary that measures should be adopted for curtailing them, provided this could be done without hardship to the deserving poor. The provident dispensary system, offered numerous advantages, but it failed to meet with general acceptance among the working class. The principle of self-help, although fully recognised at special hospitals, and at convalescent homes and cottage hospitals, throughout the country, had made very little way in the general hospitals. In referring to the difficulty of obtaining a central board empowered to deal with questions of voluntary charity, he suggested a union of the leading authorities of the Hospital Sunday Fund, who had substantial benefits to offer, and who had it in their power to secure from each hospital, on some uniform plan, a series of statistics annually, which would throw much light on many disputed questions in connection with them.

THE Academy is "full of farewells to the dying, and mournings for the dead;" certainly the picture of the year is Fildes' "The Doctor," which has the place of honour in the big gallery. It is the interior of a cottage, a sick child lies on two chairs, and leaning forward watching with grave face is the typical doctor. In the background is the father looking on with set face, the mother has laid her head down on her folded arms. Another large picture, more sentimental, less firm and truthful, is "The Crisis," by Frank Dicksee; a sick woman watched by her husband. There is a "Portrait of a Nurse," by Edward Spence, which is good—he has appreciated the simplicity of the uniform, but one could have wished for a finer face. The portrait of Sir Sydney Waterlow in the sixth gallery is very good and will be interesting to Bart.'s people. Inmates of the London will look with interest at the portrait in the eighth gallery by the wife of their Chaplain, who exhibits under her maiden name of Emma Black. There is a good portrait of Lennox Browne, one of the Rev. Frederick Pretyman, who founded the Mablethorpe Convalescent Home, one of Father Damien after the leprosy had marked him, a powerful drawing of the late Sir William Gull, a pretty likeness of Mrs. Lauder Brunton, one of Sir William Bowman, and so on. The Academy is always interesting, but it is more so than usual to hospital folk this year, partly owing to the frequent treatment of bedside scenes and to portraits, but also to the drawings in the architectural room, where there are some good designs for institution buildings. Mention must be made of Williamson's water colour, "A Poor-house Incident," which is very cleverly conceived.

THE sixth general meeting of the Hospitals Association was held at Guy's Hospital on the evening of the 29th ult., when the Treasurer of the Institution, Mr. E. H. Lushington, took the chair. The proceedings included the reading of two papers. One, by Dr. Steele, was devoted to a consideration of "Hospital Ventilation," with an account of the Victoria Infirmary at Glasgow. The second paper was read by Mr. Saxon Snell, and was "An Inquiry into the Structural Arrangements of the Nursing Departments of Hospitals." In the course of the short discussion which followed the reading of the papers, Mr. Saxon Snell, referring to Dr. Steele's paper, expressed the opinion that mechanical ventilation, though apparently good in theory, was a failure in practice.

A MEETING of the friends of the Evelina Hospital for Sick Children was held on April 22nd, when Baron Ferdinand de Rothschild presided. The report for 1890 stated that 716 children had been treated as in-patients, and 5,517 as out-patients. The Committee had been again obliged to ask the Trustees to sell out £1,000 of stock to meet current expenditure, and there still remained an amount of over £300 due to the Treasurer. The Chairman said that they would have been able to admit more cases had it not been for the want both of accommodation and of funds. He trusted they would soon be able to remedy the first want, for he had purchased a small block of adjoining houses, and when the leases fell in he hoped, with the aid of the public, to erect a new wing to the hospital. On the motion of the Hon. Conrad Dillon, seconded by Mr. F. H. Beaumont, the report was adopted. The object of the meeting was to make the institution more generally known, as at present it does not receive the amount of financial support to which it is entitled.

THE festival dinner of the North London or University College Hospital was held on the 28th ult. at the Hotel Metropole. Lord Reay occupied the chair, and proposed the toast of the evening, "Prosperity to the Hospital." In doing so he said that the year 1890 commenced with a debt of about £12,000, while the legacies received only reached the small total of £1,973. The Hospital Rebuilding Fund had remained stationary, at a sum amounting at the present time to about £12,000, and there was, therefore, a great necessity for additional subscriptions in order that the hospital might be rebuilt on a plan more in accordance with the claims of modern medical and surgical treatment. The yearly income to be relied on amounts to only about £6,000, while the expenditure approaches £20,000. The Chairman referred to the various distinguished honours gained by the members of the hospital, and urged upon all the necessity of contributing to so valuable an institution. During the evening the Treasurer's report was read, announcing subscriptions to the amount of £1,980.

A MEETING of representatives from various friendly societies was held on the 13th ult. at Bury St. Edmunds, for the purpose of considering a draft scheme with regard to the projected convalescent home for friendly societies in Suffolk. The scheme consists in the establishment of a convalescent home on the east coast by, and for the use of, members of registered friendly societies in the Suffolk district, the necessary funds being provided by subscriptions from the various societies, which will receive tickets of admission in proportion to their subscriptions. The home will be governed by a Management Committee, elected as soon as possible, and consisting of delegates elected by each of the subscribing societies, no society electing more than one delegate. It is proposed at first to start a home in a suitable house or houses, necessarily engaged for the purpose, thus avoiding the great cost of building at the outset, the means to be provided by a special inauguration fund, probably amounting to £350, which, it is hoped, will cover all preliminary and establishment expenses up to the opening of the home in 1892. This scheme has been adopted.

The Royal Hospital for Children and Women, Waterloo Bridge Road, had their festival dinner at the Hotel Metropole on Monday night, H.R.H. the Duke of Clarence and Avondale presiding. The first hospital which was firmly established for the reception of women and children, this institution has a strong claim on the charitable, a claim admitted by the City, as is shown by the subscription list; by the fact that the City of London gave a donation of 100 guineas to the dinner fund; and also by the fact that only recently the Lord Mayor gave his countenance to an appeal by the charity brought before the public at the Mansion House. Royalty, too, have always taken a great interest in the institution, to specially mark which H.M. the Queen graciously sent an extra donation of £50, and H.R.H. the Chairman, 25 guineas. Altogether subscriptions to the amount of £1,165 were announced as having been received.

Two of our metropolitan hospitals are appealing for funds to enable them to rebuild portions of their buildings, which are greatly in need of it. One of these appeals—that of the Royal Free Hospital, Gray's Inn Road—has taken the form of a festival dinner, held on the 2nd inst., at the Hotel Metropole, at which the Right Hon. the Earl of Lathom, K.G., took the chair. The Chairman was supported by the Right Hon. Lord Saye and Sele, Lord Denman, General E. Coysgame Sim, R.E., Lieut.-General Gordon Pritchard, C.B., Mr. W. W. B. Beach, M.P., Mr. Gainsford Bruce, Q.C., M.P., Mr. T. F. Halsey, M.P., and Sir George Eliot, Bart., M.P. This hospital, the Chairman pointed out, was the first to open its doors freely to the poor, letters of recommendation not being required. This, at the time (1828), was looked upon as an entire innovation, but it has since been adopted, to a greater or less extent, by other hospitals in London. What it is now sought to do is to rebuild the front of the building (at a cost of about £20,000), this being the only portion of the original building (prior to the foundation of the hospital, which was used as barracks by the Light Horse Volunteers) now in existence. The three sides of the quadrangle of which the building consists, which have been rebuilt in recent years, have been erected on the most approved principles of hospital construction, and in conformity with the rules of modern sanitary science, and this has been done without any special appeal to the public, but entirely through the liberal bequest of a former friend of the hospital. Donations, amounting in all to £8,000, were announced, £3,000 of which has come in the form of a special bequest towards the expense of rebuilding the front of the hospital.

The other hospital is the City of London Hospital for Diseases of the Chest, Victoria Park, the management of which have determined to carry out certain very necessary improvements in the drainage, sanitary appliances, and ventilation of the hospital. It has been found that the lavatories and other sanitary conveniences for the use of the patients, though conveniently situated, are cramped, inadequate, and not quite on sanitary lines as at present understood. This being so, and in order to render the hospital as healthy and up to date as possible, detached towers are to be erected at the north and south ends of the main building for the reception of proper lavatory and sanitary accommodation for all persons, both patients and staff, connected with the hospital. To carry out all this, and in order to alter the drainage where necessary, the hospital appeals for £10,000, to assist in obtaining which a meeting was held at the Mansion House on Monday last, the Right Hon. the Lord Mayor in the chair. Leaving out all other considerations, seeing the lack of beds for the class of patients treated in this hospital, which exists in the metropolis, it is essential that those consumption hospitals which we do possess (and they are only four in number) should be all as thoroughly perfect in construction and sanitation as it is possible to make them, and we therefore congratulate the Committee on their having taken this important step, and hope that they will obtain all the aid they ask for. Subscriptions from Her Majesty the Queen (£50) and H.R.H. the Duke of Connaught (25 guineas) were announced.

